# Assessing Mood With the Identifying Depression Early in Adolescence Chatbot (IDEABot): Development and Implementation Study

**DOI:** 10.2196/44388

**Published:** 2023-08-07

**Authors:** Anna Viduani, Victor Cosenza, Helen L Fisher, Claudia Buchweitz, Jader Piccin, Rivka Pereira, Brandon A Kohrt, Valeria Mondelli, Alastair van Heerden, Ricardo Matsumura Araújo, Christian Kieling

**Affiliations:** 1 Department of Psychiatry Universidade Federal do Rio Grande do Sul Porto Alegre Brazil; 2 Child and Adolescent Psychiatry Division Hospital de Clínicas de Porto Alegre Universidade Federal do Rio Grande do Sul Porto Alegre Brazil; 3 Center for Technological Advancement Universidade Federal de Pelotas Pelotas Brazil; 4 Social, Genetic & Developmental Psychiatry Centre Institute of Psychiatry, Psychology & Neuroscience King’s College London London United Kingdom; 5 Economic and Social Research Council Centre for Society and Mental Health King’s College London London United Kingdom; 6 Division of Global Mental Health Department of Psychiatry, School of Medicine and Health Sciences The George Washington University Washington, DC United States; 7 Department of Psychological Medicine Institute of Psychiatry, Psychology King’s College London London United Kingdom; 8 Centre for Community Based Research Human Sciences Research Council Pietermaritzburg South Africa

**Keywords:** depression, adolescent, ambulatory assessment, chatbot, smartphone, digital mental health, mobile phone

## Abstract

**Background:**

Mental health status assessment is mostly limited to clinical or research settings, but recent technological advances provide new opportunities for measurement using more ecological approaches. Leveraging apps already in use by individuals on their smartphones, such as chatbots, could be a useful approach to capture subjective reports of mood in the moment.

**Objective:**

This study aimed to describe the development and implementation of the Identifying Depression Early in Adolescence Chatbot (IDEABot), a WhatsApp-based tool designed for collecting intensive longitudinal data on adolescents’ mood.

**Methods:**

The IDEABot was developed to collect data from Brazilian adolescents via WhatsApp as part of the Identifying Depression Early in Adolescence Risk Stratified Cohort (IDEA-RiSCo) study. It supports the administration and collection of self-reported structured items or questionnaires and audio responses. The development explored WhatsApp’s default features, such as emojis and recorded audio messages, and focused on scripting relevant and acceptable conversations. The IDEABot supports 5 types of interactions: textual and audio questions, administration of a version of the Short Mood and Feelings Questionnaire, unprompted interactions, and a *snooze* function. Six adolescents (n=4, 67% male participants and n=2, 33% female participants) aged 16 to 18 years tested the initial version of the IDEABot and were engaged to codevelop the final version of the app. The IDEABot was subsequently used for data collection in the second- and third-year follow-ups of the IDEA-RiSCo study.

**Results:**

The adolescents assessed the initial version of the IDEABot as enjoyable and made suggestions for improvements that were subsequently implemented. The IDEABot’s final version follows a structured script with the choice of answer based on exact text matches throughout 15 days. The implementation of the IDEABot in 2 waves of the IDEA-RiSCo sample (140 and 132 eligible adolescents in the second- and third-year follow-ups, respectively) evidenced adequate engagement indicators, with good acceptance for using the tool (113/140, 80.7% and 122/132, 92.4% for second- and third-year follow-up use, respectively), low attrition (only 1/113, 0.9% and 1/122, 0.8%, respectively, failed to engage in the protocol after initial interaction), and high compliance in terms of the proportion of responses in relation to the total number of elicited prompts (12.8, SD 3.5; 91% out of 14 possible interactions and 10.57, SD 3.4; 76% out of 14 possible interactions, respectively).

**Conclusions:**

The IDEABot is a frugal app that leverages an existing app already in daily use by our target population. It follows a simple rule-based approach that can be easily tested and implemented in diverse settings and possibly diminishes the burden of intensive data collection for participants by repurposing WhatsApp. In this context, the IDEABot appears as an acceptable and potentially scalable tool for gathering momentary information that can enhance our understanding of mood fluctuations and development.

## Introduction

### Background

The challenges and limitations of the current tools of mental health assessment—mostly performed using standardized scales—have increased the interest in alternative monitoring tools. Traditional assessment often fails to incorporate the dynamic nature of psychological constructs and other relevant clinical features [1] and is not capable of capturing prognostic and therapeutic differences among patients [2] as well as the personalized aspects that are essential to address mental health issues.

Over recent decades, technology has created an opportunity to expand data collection and analysis beyond clinical and research facilities and centers, with flexibility to create participative, 2-way communication applications that can be easily adapted and used in everyday settings for a variety of target populations [3]. Considering the central role of language in the diagnosis and assessment of mental health, a shift toward a technology focused on conversational aspects may be key to systematizing natural language domains that are not currently explored in clinical settings [4].

In this sense, we propose that using chatbots—digital systems that rely on a conversational interaction that mimics human conversation [5]—may be an alternative to using traditional assessment methods. Chatbots are capable of capturing real-time accounts of events (ie, at the moment the event is being experienced) [6] and thus may further our current understanding of time- and context-contingent associations among activities, moods, and experiences [7]. Primarily, it has been theorized that chatbots both facilitate disclosure [8,9] and provide an opportunity to collect real-time information on mood and behavior in real-world settings with lower perceived burden for participants and researchers, increasing ecological validity, minimizing recall biases [10], and taking advantage of human-like conversation features to assess psychological constructs (such as *depression*) in a scalable, systematic fashion that is not possible with the usual application of instruments and scales.

One important advantage of chatbots is that they may be integrated into existing applications that are routinely used by the general public and designed as affordable, potentially scalable tools, following a frugal innovation model [11]. In addition, chatbots could be explored to reduce barriers that typically prevent identification of mental health disorders among, and help-seeking by, young people, a group especially susceptible to these conditions [12]. Given the scarcity of resources allocated to mental health care, particularly in middle-income countries such as Brazil, the development of frugal chatbot apps is a promising alternative.

### Objectives

Chatbots have been used in mental health research for purposes such as therapy, training, and screening [13,14]. Nevertheless, most studies on user-chatbot interactions have focused on adults [15], although adolescents are often more familiar with smartphones than other populations [16]. Thus, exploring the feasibility of using chatbots to collect data on adolescent mood and behavior in an ecological fashion may be a promising avenue of inquiry. We hypothesize that, by leveraging already existing technologies, chatbots are a feasible, viable form of monitoring changes in mood and symptoms over time in adolescent populations. Moreover, we believe that their use lessens participant burden, possibly augmenting sustained engagement with the tool.

Therefore, we aimed to develop a chatbot tool to collect real-life data on mood and behavior from adolescents using text and audio messages. Here, we present the development and feasibility pilot of and initial results obtained with the implementation of the WhatsApp-based Identifying Depression Early in Adolescence Chatbot (IDEABot).

## Methods

### Study Setting: Identifying Depression Early in Adolescence Risk Stratified Cohort

The IDEABot was developed as part of the Identifying Depression Early in Adolescence Risk Stratified Cohort (IDEA-RiSCo) study [17]. The IDEA-RiSCo study includes 150 Brazilian adolescents (n=75, 50% female participants and n=75, 50% male participants) aged 14 to 16 years at baseline, stratified into 3 groups: low risk for developing depression (50/150, 33.3%), high risk for developing depression (50/150, 33.3%), and experiencing a current untreated major depressive episode (50/150, 33.3%). Participants were selected for each group using the Identifying Depression Early in Adolescence Risk Score (IDEA-RS), an empirically generated algorithm developed to estimate the individual-level probability of a unipolar depressive episode 3 years after initial assessment [17-19]. Additional details on procedures used in the IDEA-RiSCo study are described elsewhere [17].

### Rationale and Feasibility Pilot

The IDEABot was developed to collect data from Brazilian adolescents via WhatsApp (Meta) [11]. In 2019, WhatsApp was reported to have been used at least once every hour by 81% of Brazilians [20]. Moreover, among adolescents from public state schools in the city of Porto Alegre, Rio Grande do Sul, Brazil (the population from which the IDEA-RiSCo sample was derived), WhatsApp was the most popular web-based platform, used at least once a day by 90% of the sample [21].

The IDEABot was devised to collect daily data on current mood via both structured items or questionnaires and free audio reporting of the aspects of daily life considered by participants (Multimedia Appendix 1). An interdisciplinary team was engaged in the project, including mental health practitioners (psychiatrists and psychologists), computer scientists, and writers. The prototype version of the IDEABot was designed and implemented in Brazilian Portuguese using inputs from the research team, followed by a feasibility pilot that generated a round of adjustments.

For the feasibility pilot, 6 adolescents were invited to test a prototype version of the IDEABot and comment on their user experience. They tested the chatbot system for 5 days, during which they answered the Short Mood and Feelings Questionnaire (sMFQ) and participated in 2 days of brief audio recordings. All features and possible response modes were tested. After test completion, the adolescents participated in an individual interview and a focus group discussion, conducted on the web by 2 researchers (AV and CK).

The interviews focused on the overall experience, feasibility, and acceptability of using the IDEABot (including concerns about data safety and privacy). In addition, the adolescents were engaged in jointly exploring and proposing improvements and solutions for perceived problems. In the focus group, anchored vignettes were used [22] to explore participants’ perceptions of the chatbot (Multimedia Appendix 2).

### Implementation of the Final Version of the IDEABot

After the pilot test, the final version of the IDEABot was generated and subsequently implemented in the second- and third-year follow-ups of the IDEA-RiSCo study [17]. On the basis of a review of the literature, the following usability indicators [23] were evaluated to define successful implementation [24,25]: (1) acceptance (ie, the proportion of participants who were invited to take part in the IDEABot data collection and agreed to use the tool); (2) initial attrition (ie, failure to further engage in the protocol after agreeing to participate in the data collection and complete the initial steps); and (3) compliance, defined as the proportion of days on which participants generated at least 1 data point over the 15 days of data collection.

Socioeconomic status was also assessed with data collected at baseline using the Brazilian Criterion of Economic Classification [26], along with administration of a 9-item questionnaire on the frequency of the participants’ use of 8 social media platforms, including the frequency of WhatsApp use [21,27]. Responses were aggregated into 3 strata (1=never, 2=several times/week, and 3=several times/day or constantly).

Categorical and numerical variables were compared using the chi-square and Mann-Whitney *U* tests, respectively. In addition, the Spearman correlation coefficient was used to verify correlations among continuous variables. All analyses were performed using SPSS software (version 26.0; IBM Corp).

### Ethics Approval

The development and research use of the IDEABot was approved by the Hospital de Clínicas de Porto Alegre ethics committee (50473015.9.0000.5327).

### Informed Consent and Participation

All adolescents and caregivers provided written assent or consent to participate in each stage of data collection and were given the opportunity to withdraw assent or consent at any time. For participants aged >18 years, written consent was obtained directly. If participants wished to stop receiving messages from the chatbot before the completion of the 15-day trial, they were instructed to contact a research team member. In addition, participants were instructed to use the WhatsApp *delete* button if they preferred to delete sent messages or audio files. Along with the research team’s explanation on the functioning of the IDEABot, the chatbot’s first interaction with the user explicitly stated the nature of the exchange that would take place. Participants were thus aware that the audio recordings were not listened immediately and that the chatbot was not a channel for seeking help. Participants were provided with an additional telephone number and instructed to contact a team member (a board-certified psychiatrist) in case they were actively seeking information related to mental health issues. Furthermore, participants received information regarding the national helpline for health and safety emergencies. Following Brazilian legislation, participants did not receive financial incentives for taking part in the study but were offered compensation for mobile internet data use during their participation.

## Results

### Results of the Feasibility Pilot

Six adolescents (n=4, 67% male participants and n=2, 33% female participants; n=1, 25% of the 4 male participants had lived experience of depression, as did n=1, 50% of the 2 female participants) aged 16 to 18 years participated in the feasibility pilot. They were selected by convenience among the group of adolescents who had already participated in other projects conducted by our research team. Despite their heterogeneous socioeconomic backgrounds, all had a smartphone with internet access. Parental consent was obtained for all underage participants (those aged <18 years). As most of the participants (5/6, 83%) had already participated in other stages of the research, they were familiar with the investigators and knew about the IDEA-RiSCo objectives and procedures. The interviews lasted 20 to 30 minutes, whereas the duration of the focus group was 50 minutes.

Overall, participants considered the IDEABot easy to use and enjoyable. All 6 adolescents completed at least 4 (80%) of the 5 interactions and sent an average of 54.5 (range 2-97) seconds of audio recordings per day. The adolescents expressed that directed questions (such as those asking about their daily routine) were easier to answer than more open questions (such as the initial request for participants to introduce themselves). In addition, the adolescents considered the prompts that targeted the collection of at least 1 minute of audio recordings over the day to be adequate.

Overall, they perceived the burden of integrating the chatbot into their daily routine as low. In fact, they highlighted a positive effect of talking about their daily lives:

It was a good experience...I felt I was talking about my things to someone—it even sounded like there was someone there wanting to know how my day was. Sometimes you spend your day without anyone asking you that. But the chatbot asked.

Female participant, aged 17 years

Regarding the sMFQ, the adolescents found that some of the instructions provided by the chatbot were unclear and made suggestions on how to fix these issues. It asked participants to answer the sMFQ using the numbers 0, 1, or 2. The adolescents suggested further anchoring of these responses (eg, through reminders of the meaning of each number during the completion of the questionnaire). The instructions were adjusted accordingly after these difficulties and possible solutions were explored with the adolescents. In the final version, an explanation of each possible choice of answer was provided (*0*=no, *1*=sometimes, and *2*=yes) before the participants were asked to complete each item of the sMFQ, using, for example, the statement “I feel sad today.” In addition, a short reminder of the meaning of each numeric answer (0, 1, or 2) was added after each chatbot prompt.

An important adjustment made possible by the feasibility pilot was as follows: the adolescents tended to respond to the chatbot’s final interaction by either thanking it or sending an emoji. In the chatbot’s initial programming, this was interpreted as an unsolicited interaction to which the IDEABot responded by requesting an audio message to explain what the participant had said. This chatbot response would often confuse the adolescents. To avoid this, we developed a content-based rule: if participants responded with a predefined set of words (“ok,” “see you,” “thank you,” or variations), this was interpreted as a conversation closure, and the chatbot’s probe would not be triggered.

Another aspect that required changing was suggested by the adolescents in relation to the schedule of interactions. The adolescents argued that they would most likely be at school or asleep at 10:30 AM and therefore would probably not feel comfortable responding to the questions owing to their current environment (especially if they were at school). The adolescents then suggested that the first interaction of the day be moved from 10:30 AM to 1:30 PM, which was implemented in the final version of the IDEABot.

### Implementation of the IDEABot

#### Development of the IDEABot

The IDEABot was successfully developed to perform prescripted interactions requesting audio and text responses from participants to the questions it posed. The chatbot questions and responses were expressed only in text format, regardless of the format of user input. The IDEABot was also designed to delay answers proportionally to the length of the text being sent to users to simulate a more natural typed conversation. Using a rule-based approach, four types of interactions were developed: (1) mood ratings, (2) emoji mood ratings, (3) brief audio recordings, and (4) questionnaire answers (Multimedia Appendix 3).

As a first step to activate the chatbot, users were required to send a WhatsApp text message (any content was acceptable) to the chatbot’s mobile number. To ensure both the standardization of instructions given to users and clarity regarding the nature of the conversation, as well as to prevent misconceptions (such as participants believing that the chatbot is a real person or that the audio recordings would be listened immediately), the first interaction with the chatbot was designed to review overall functionality. This initial interaction was named *day 0* and covered the routines that users should expect over the subsequent 14 days and how they were supposed to respond. Because of the IDEABot’s nature and objective, data generated on day 0 will be excluded from future analyses.

**Figure 1:**
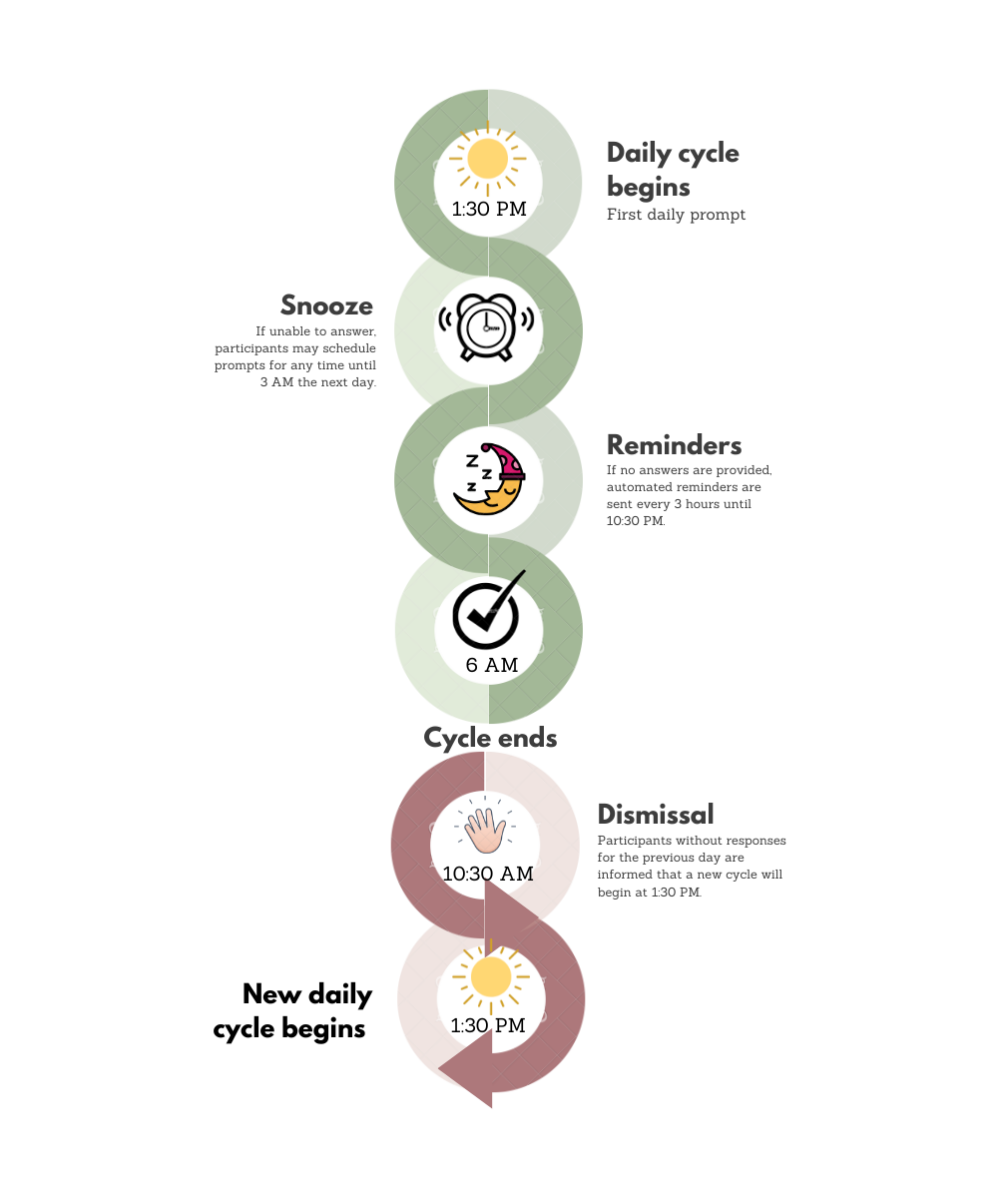
Overview of the functioning of the Identifying Depression Early in Adolescence Chatbot over the period of a day.

The chatbot follows a time-contingent sampling for each participant. In this sense, it is designed to initiate interactions at fixed times: every day, beginning at 1:30 PM, participants receive a message asking whether they are available to answer the scheduled questions. They may answer immediately after the first message prompt or use a *snooze* function to schedule a reminder for a later time in the day (the IDEABot allows snoozing until 3 AM the next day). If participants ignore the first prompt, additional messages are sent at 3-hour intervals. Participants have until 6 AM the following day to respond to the questions of each daily cycle. If the interaction is not completed, at 10 AM the following day, the chatbot informs the participant that the daily cycle will end without completion and that a new daily cycle will begin, also providing the time when the next message would be sent. In addition to scheduled interactions, participants are also given the option to send unprompted audio recordings throughout the day (Figure 1).

**Figure 2:**
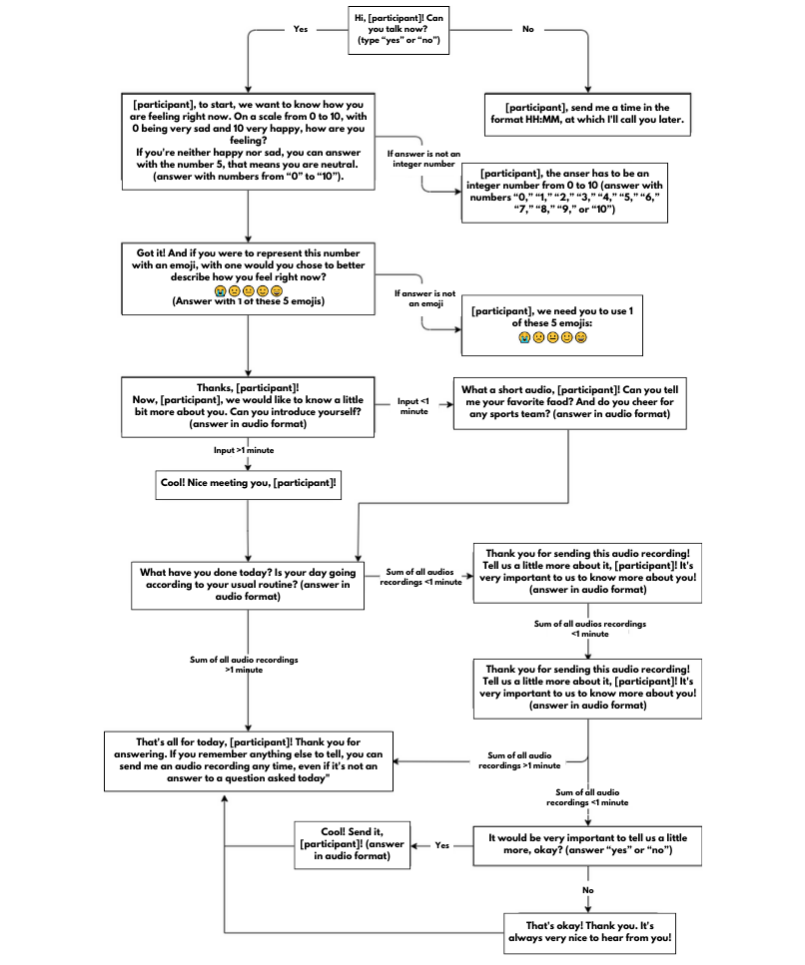
Example of the interaction with users of the Identifying Depression Early in Adolescence Chatbot: day 2.

The chatbot’s schedule is divided into five interaction modes: (1) introduction (the first interaction with users), (2) audio questions, (3) administration of a version of the sMFQ, (4) other messages, and (5) the *snooze* function (Multimedia Appendix 3). On 7 (47%) of the 15 days, IDEABot asks broad questions about daily life, social interactions, and preferences (Textbox 1), and participants are invited to answer through audio recordings. The goal is to collect at least 1 minute of audio recordings per day from each participant. If the answers provided by participants to the 2 daily questions do not add up to 1 minute in duration, the chatbot asks 2 standard follow-up questions, encouraging the participant to say more. If after the first follow-up question (“Thank you for sending this audio! Tell us a little bit more about it, [participant]!”), the total duration of the audio recording still does not reach 1 minute, the chatbot sends the second question (“It would be very important if you could tell us a little more, okay?”). Regarding this last question, participants can choose whether to send another audio recording (typing “yes” or “no” before sending the audio recording). One example is provided in Figure 2.

On the 7 days without audio prompts, participants are asked to complete the sMFQ [28,29]. The 13 questions of the sMFQ cover the current day (instead of the last 2 weeks as in the original sMFQ; Multimedia Appendix 4). Participants are instructed to type 0, 1, or 2 to answer each question, and they have the option to correct their answers (for relevant aspects of the processing of the collected data and analyses, refer to Multimedia Appendix 5 [30-36]).

**Questions (question 1 [Q1] and question 2 [Q2]) or prompts for audio responses requested by the Identifying Depression Early in Adolescence Chatbot (the original questions are in Brazilian Portuguese).**

**Day 1**

- Q1. Can you introduce yourself?
- Q2. What have you done today? Is your day going according to your usual routine?

**Day 3**

- Q1. Are you at home?
  [If the response is “yes”] What are you doing? Is someone else around?
  [If the response is “no”] Who do you live with? Do you get along with the people you live with?
- Q2. Can you tell me more about your house? Do you like living there?

**Day 5**

- Q1. Did you go outside today at all, [participant]? Do you spend more time inside, or do you sometimes go out? When you’re out, what do you normally do?
- Q2. And how’s your neighborhood? Are there nice things around?

**Day 7**

- Q1. Today I want to know about your favorite story. What is it? You can choose a movie, a series, a book...whatever you want!
- Q2. And why is this your favorite story, [participant]?

**Day 9**

- Q1. Do you use your mobile phone a lot, [participant]? What are your favorite things to do on the mobile phone?
- Q2. And how much time do you think you spend on the internet each day? Do you use the internet mostly during the day or at night? Why?

**Day 11**

- Q1. Not counting the audio recordings you send here [*grinning face with sweat* emoji], who do you talk to about things that happen in your life? How’s your relationship with this person?
- Q2. And why do you trust this person?

**Day 13**

- Q1. It’s been almost 2 weeks since we started talking, [participant]! How did you feel about answering these questions?
- Q2. And how have you been in these last 2 weeks? Has anything different happened?

#### Initial Results of a Full-Sample Implementation of the IDEABot

The IDEABot was first implemented as part of the IDEA-RiSCo second-year follow-up assessment, which took place between August 1, 2020, and January 31, 2022. It was subsequently also used in the third-year follow-up of the IDEA-RiSCo sample, which occurred between August 1, 2021, and September 30, 2022.

To explain the chatbot’s functioning and features to participants, an animated video (Multimedia Appendix 6) was developed by the research team, providing a comprehensive overview of the research process. It reminded participants about the previous waves of data collection and the overall research goal, as well as presented the various steps of data collection that they could engage in (including the IDEABot). In addition, the video provided information regarding data confidentiality, including end-to-end encryption by WhatsApp for all chats, and the measures taken by the research team to ensure data protection. After the video was sent, if participants agreed to use the IDEABot, a research team member sent a link that directed users to initiate the interaction.

For the second- and third-year follow-up assessments, 9.7% (11/113) and 11.5% (14/122) of the adolescents, respectively, did not have a smartphone and agreed to receive a device from the study team to enable data collection completion. All other participants used their own smartphones and already had WhatsApp installed. In terms of technical challenges experienced during the IDEABot implementation, we recorded 6 and 14 occurrences or technical malfunctions in the second- and third-year follow-up assessments, respectively.

In the second-year follow-up, there were 5 issues with the integration with WhatsApp’s application programming interface (API; September 11 and 15, 2020; December 11, 2020; April 4, 2021; and June 15, 2021) and 1 instance in which WhatsApp was offline around the world owing to an instability in Meta’s servers (October 4, 2021) [37]. All issues were resolved within 24 hours, but the interactions of 6.2% (7/113) of the participants were affected directly. As result, these participants lost 8 interaction days in total. In addition, in the second-year follow-up, there were 3 instances in which the chatbot’s malfunctioning prevented participants from completing the scheduled interactions. In all cases, participants repeated the interaction days affected. Finally, there was 1 occasion on which a participant was not able to complete the day’s interaction owing to a problem with telephone billing, which was later resolved.

In the third-year follow-up, there were 12 issues with the integration with WhatsApp’s API (March 18 and 19, 2022; April 5 and 20, 2022; May 5, 18, and 20, 2022; June 14 and 26, 2022; July 8, 2022; and August 16 and 28, 2022), as well as 2 instances in which chatbot was unable to access the internet (October 10, 2021, and February 18, 2022). In addition, the instance in which WhatsApp was offline worldwide (October 4, 2021) also affected the third-year follow-up. Only 1 occurrence was not resolved within 24 hours (March 18 and 19, 2022), owing to the API’s instability. Interactions were affected for 33.6% (41/122) of the participants, resulting in a loss of 16 occasions in which these participants could have completed the day’s interaction. The greatest number of occurrences were mostly caused by the changes in WhatsApp Web, the web-based interface for WhatsApp required for running the API.

In the second-year follow-up, 140 adolescents took part in some aspects of data collection and were therefore eligible to use the IDEABot. Of the 140 adolescents, 113 (80.7%) agreed to use the IDEABot and completed the initial interaction. Of these 113 participants, 1 (0.9%) interacted with the chatbot only on the first interaction. The 112 adolescents who continued interacting with the chatbot engaged on average 12.8 (SD 3.5) of the 14 possible days, corresponding to a compliance rate of 91.4%. The *snooze* function was used 609 times, resulting in 331 completed interactions. In addition, participants sent on average 65 (SD 37.7) seconds of audio recordings per day, resulting in an average of 7.6 (SD 4.3) minutes of audio recordings per participant.

For the third-year follow-up, 132 adolescents took part in some aspects of data collection and were therefore eligible to use the IDEABot. Of the 132 adolescents, 122 (92.4%) agreed to use the IDEABot and completed the initial interaction. Of these 122 participants, 1 (0.8%) interacted with the chatbot only on the first interaction. The 121 adolescents who continued interacting with the chatbot engaged on average 10.57 (SD 3.4) of the 14 possible days, corresponding to a compliance rate of 75.5%. The *snooze* function was used 569 times, resulting in 258 completed interactions. In addition, participants sent an average of 69.2 (SD 66.1) seconds of audio recordings per day, resulting in an average total of 8.1 (SD 7.8) minutes of audio recordings per participant.

No significant association between socioeconomic status and the number of days of interaction with the IDEABot was found (*P*.88); the number of days on which responses were recorded also did not differ when participants were stratified according to the pattern of previous WhatsApp use (ie, never, several times/week, or several times/day; *P*.98) or by sex (male or female; *P*.66).

## Discussion

### Principal Findings

This study outlines the development, feasibility pilot, and initial results obtained with the implementation of a chatbot to support mood assessment in adolescents. Although chatbots are becoming increasingly more common in health care settings [38], few studies have provided detailed analyses and empirical discussions of specific design elements and development techniques [39]. In this sense, we believe that reporting the development and implementation of the IDEABot is a novel and relevant contribution, especially given the overall good acceptance for using the tool, low attrition, and high compliance in terms of the proportion of responses in relation to the total number of elicited prompts.

To the best of our knowledge, the IDEABot is the first chatbot specifically tailored to aid multimodal research data collection with adolescent populations. Our decision to use an existing platform made it possible to design, develop, and implement the IDEABot in a way that directly addresses the constraints that the use of new mobile apps may pose to research teams and users, in addition to saving development and adjustment time. The IDEABot runs on any smartphone with WhatsApp, regardless of operating system, as long as internet connectivity is available. The IDEABot thus qualifies as a frugal innovation: it is significantly cheaper than other alternatives (such as the development of a new stand-alone app); it has proven sufficient for the proposed level of data collection; and by using it, we were able to reach participants who would otherwise remain underrepresented [11]. Moreover, the proposed approach to data collection is highly flexible and could potentially leverage all forms of interactions available on WhatsApp, including photographs and video recordings.

The initial administration of the IDEABot indicates engagement rates of >80%, with more than half of the participants (59/113, 52.2% and 52/122, 42.6% for second- and third-year follow-up use, respectively) completing all 15 days of collection. In ecological momentary assessment studies (ie, studies that are designed to collect individual data at several time points), 80% has been proposed as an indicator of adequate compliance [40]. Although compliance tends to vary in ecological momentary assessment studies (also depending on the number of measures made over time) [41], we believe that the rate obtained with the IDEABot matches the expected rates in similar studies and is adequate, considering the target population and that no financial or other direct incentive was used.

In this sense, we believe that repurposing an already ubiquitous tool in the life of adolescents to collect research data can increase overall engagement as well as diminish the perceived burden of data collection. Moreover, we highlight the importance of youth participation in the creation, adaptation, and implementation of the IDEABot. A chatbot’s personality, interaction flow, conversation length, and dialogue structure are important aspects and can influence user satisfaction [39]. In the case of the IDEABot, all these aspects were created and tailored with the aid of a group of adolescents, who were active in pointing out any strangeness or discomfort and were ready to brainstorm solutions. Thus, not only was the final chatbot tailored to collect relevant research data, but it was also pleasant in terms of appearance and the manner of interaction with adolescents themselves, which can greatly decrease the burden of research participation.

All things considered, the IDEABot still has important limitations that need to be addressed. Despite good engagement rates among Brazilian adolescents, the IDEABot is a basic chatbot that uses a rule-based approach. Although this gives the researchers optimal control over conversation flow and topics, the limited response range may decrease usability by adolescents (who may, for example, become frustrated with repeated error messages) [42]. In addition, as a WhatsApp-based chatbot, the IDEABot is susceptible to changes in policies and bugs affecting the platform. In this sense, the usability of the IDEABot becomes heavily linked to WhatsApp as a commercial product, and researchers have no control over policies such as data security and other features. The instance in which WhatsApp was offline worldwide preventing data collection is also an indication of the bot’s susceptibility to the platform’s functioning, which may hinder its applicability.

Furthermore, although the chatbot’s user-oriented design may contribute to higher self-disclosure [43], privacy concerns regarding the use of the data are a relevant topic. WhatsApp policies include “end-to-end encryption” [44], and the IDEABot also stores information (audio recordings and conversation logs) on secure encrypted servers with additional anonymization of sensitive information in reports. However, all conversation logs and sent audio files remain accessible to other users in the mobile phone or any other devices that may be used to connect to WhatsApp (such as WhatsApp Web). Local backups may also store this information in user’s mobile phones, creating the risk of confidentiality breaches that cannot be controlled by the research team.

Another important aspect is the chatbot’s response to serious health concerns. As the IDEABot often queries participants on mood and daily events, we might expect sensitive information to be disclosed at the moment when distressing events occur. However, the IDEABot’s rule-based approach may not be suitable for fully and effectively responding to these events. In our project, mitigation efforts included full disclosure that audio messages would not be listened to immediately by the research team and that the IDEABot was not equipped to deal with mental health emergencies. Participants were also provided with the national emergency service hotline number for acute cases, and they were also able to call a research team psychiatrist in case of significant distress during the data collection process. However, this particular safety measure was never used by participants during the data collection process in either follow-up wave.

Also important is the susceptibility of the interface to technical error, such as bugs in the chatbot response routine (it does not respond, or it provides responses that do not fit the conversation context). As people may anthropomorphize chatbots [43], perceiving them as having a mind with intention, consciousness, and goals [45], these instances may generate negative feelings or distress responses, with a potential negative impact on participants who could become attached to the chatbot [46], or even hinder retention and continuous use. For the IDEABot, preventive measures include continuous function supervision by both humans and software monitoring the integration with WhatsApp’s API. In addition, using the platform as a medium for data collection also gives researchers little control over the quality of the data while they are being collected. This can be critical, for example, during data analysis, in which the selection, extraction, and assessment of acoustic features are dependent on the quality of the audio files and the data obtained [30]. This highlights the need for further research to explore the data collected as well as the techniques that are best suited for collecting and analyzing the data.

Therefore, the IDEABot presents limitations that may be considered inherent to the methods chosen. However, its development was guided by the principle of user transparency, and challenges regarding privacy and adverse incidents have been, and continue to be, closely and continuously assessed throughout development, implementation, and use. In addition, we believe that, as a tool, the IDEABot supports stakeholder values [47]. Nonetheless, the ethical considerations involving chatbot use will change with time and technical development, and continuous reassessment is vital to address any resulting ethical concerns that may arise.

### Conclusions

The IDEABot is a novel WhatsApp chatbot developed to aid intensive longitudinal collection of mood data among adolescents. The collection of audio recordings and information on mood and behavior throughout 15 days may enable analyses of adolescents’ data that would otherwise not be possible. The completion rate shows that the IDEABot was able to collect information in a manner that is attuned to the adolescents’ lives. In this sense, the use of sequenced audio recordings may be considered similar to an audio diary, capturing much of the sense making and representation of experiences at different time points [48].

It is worth noting that the choice for a multimodal data collection approach that combines audio recordings of prompted speech, daily information on mood, and traditional assessment methods (such as questionnaires) sheds light on aspects of depression—such as the temporal evolution of symptomatology—that have only recently become a focus of research and are also rapidly advancing. Thus, the IDEABot generates a rich database that combines different types of input information that can be compared and triangulated.

The IDEABot is a frugal innovation and therefore has a goal to meet the basic needs of a population that would otherwise remain underserved [11]; a strength of the IDEABot is its reliance on an available ubiquitous medium as a way to reach a population that is still underrepresented in research [49,50]. However, adaptability is key, and thus we chose to use a simple rule-based approach, allowing the IDEABot to be easily implemented, both technically and economically. As a result, the IDEABot is a feasible tool for data collection that can be adapted, tested, and implemented in different settings and for different purposes.

Another strength of the IDEABot is its capability for intensive data collection over extended periods within a longitudinal 3-year research project with a careful phenotypic characterization of the sample, including multiple informants. Such intensive and momentary data collection can elucidate aspects of the overall trajectory of different groups of individuals, such as those taking part in the IDEA-RiSCo study. This group approach can be useful for monitoring change and fluctuations in mood and to address the overall trajectories of different groups over time. In addition, periods of intensive data collection in individual participants may capture unique changes or symptom fluctuation patterns that would not otherwise be detected [7], contributing important information regarding symptom connectivity and centrality over time. The contrast between group and idiographic findings provides a further level of information not usually available in traditional research designs. In this sense, in addition to furthering our understanding of individual and group trajectories, the characterization of the sample also provides an opportunity to further explore the patterns of chatbot-assisted data collection.

In summary, the initial apps of the IDEABot were successful. The IDEABot seems to be a feasible, potentially scalable tool to collect data that can further our understanding of how mood changes and develops over time among adolescents.

## Appendix

Multimedia Appendix 1 Technical aspects of the development of the Identifying Depression Early in Adolescence Chatbot (IDEABot).

Multimedia Appendix 2 Anchoring vignettes.

Multimedia Appendix 3 The types of interactions users can have with the Identifying Depression Early in Adolescence Chatbot (IDEABot).

Multimedia Appendix 4 Chatbot script for the Short Mood and Feelings Questionnaire instructions.

Multimedia Appendix 5 Processing and analysis of data and questionnaires.

Multimedia Appendix 6 Animated video.

## Abbreviations

API: application programming interface
IDEABot: Identifying Depression Early in Adolescence Chatbot
IDEA-RiSCo: Identifying Depression Early in Adolescence Risk Stratified Cohort
IDEA-RS: Identifying Depression Early in Adolescence Risk Score
sMFQ: Short Mood and Feelings Questionnaire
