# Supplementary material for: Assessing Mood With the Identifying Depression Early in Adolescence Chatbot (IDEABot): Development and Implementation Study
Source: JMIR Hum Factors. 2023 Aug 7;10:e44388. doi: 10.2196/44388 (PMC10442728; doi:10.2196/44388)
Supplement: Multimedia Appendix 1 [file humanfactors_v10i1e44388_app1.docx]

**Supplementary file for “Assessing Mood With the Identifying Depression Early in Adolescence Chatbot (IDEABot): Development and Implementation Study”**

**Multimedia Appendix A** - Technical aspects of the development of the IDEABot

IDEABot was implemented in WhatsApp using NodeJs, a JavaScript runtime to build general purpose applications. Because the chatbot was designed with a rule-based approach, interactions are modeled via a state machine. When participants interact with the bot, responses are selected depending on multiple conditions, such as the current script step, the participant’s response, time of day, or total duration of audio messages sent during each daily cycle. Additionally, textual responses are checked for correctness (format and range of accepted values). If participants provide an incorrect answer, the IDEAbot engine repeats the question using a pre-scripted error message.

Written communication with the chatbot is matched against a predefined set of possible response/exchange categories. These categories include: greetings (all moments when the bot initiates an interaction, often saying ‘hello’ to participants), Yes/No-type answers, the snooze function, or a numerical answer. Each category has a corresponding regular expression to match user input. Depending on the current conversation state, time of day, and the matching category, IDEABot sends a predetermined answer and updates the conversation state. In addition to parsed information such as the category of the answer, the raw texts from the user and chatbot are also stored.

User state is stored in a relational database in which data are queried for analysis. User current state and conversation progress is maintained in a PostgreSQL database (<https://www.postgresql.org>). Text answers and audio metadata such as duration and corresponding prompt are also stored in this database. All information collected by IDEABot — audio files, conversation logs and answers provided to questionnaires — is protected by the WhatsApp encryption and subsequently uploaded to our own secure server. Audio messages are always saved but only linked to a specific prompt from the chatbot when sent at the appropriate moment in the conversation. Audio data is uploaded to Dropbox via its developer API (<https://www.dropbox.com/developers/>) and stored locally on a local server for redundancy purposes.
