# Supplementary material for: Assessing Mood With the Identifying Depression Early in Adolescence Chatbot (IDEABot): Development and Implementation Study
Source: JMIR Hum Factors. 2023 Aug 7;10:e44388. doi: 10.2196/44388 (PMC10442728; doi:10.2196/44388)
Supplement: Multimedia Appendix 2 [file humanfactors_v10i1e44388_app2.docx]

**Supplementary file for “Assessing Mood With the Identifying Depression Early in Adolescence Chatbot (IDEABot): Development and Implementation Study”**

**Multimedia Appendix B** - Anchoring vignettes

Anchored vignettes have been used to assess domains of technology acceptability and feasibility in settings with potentially high social desirability bias such as with adolescents and in low- and middle-income countries. In our study, we created six anchoring vignettes in Brazilian Portuguese. Each vignette showed the reaction of two fictional characters, Luís and Pedro, to six features of IDEABot. Adolescents were asked to discuss the perspectives of both characters and choose their preference.

Scenario 1 - Ideal audio duration


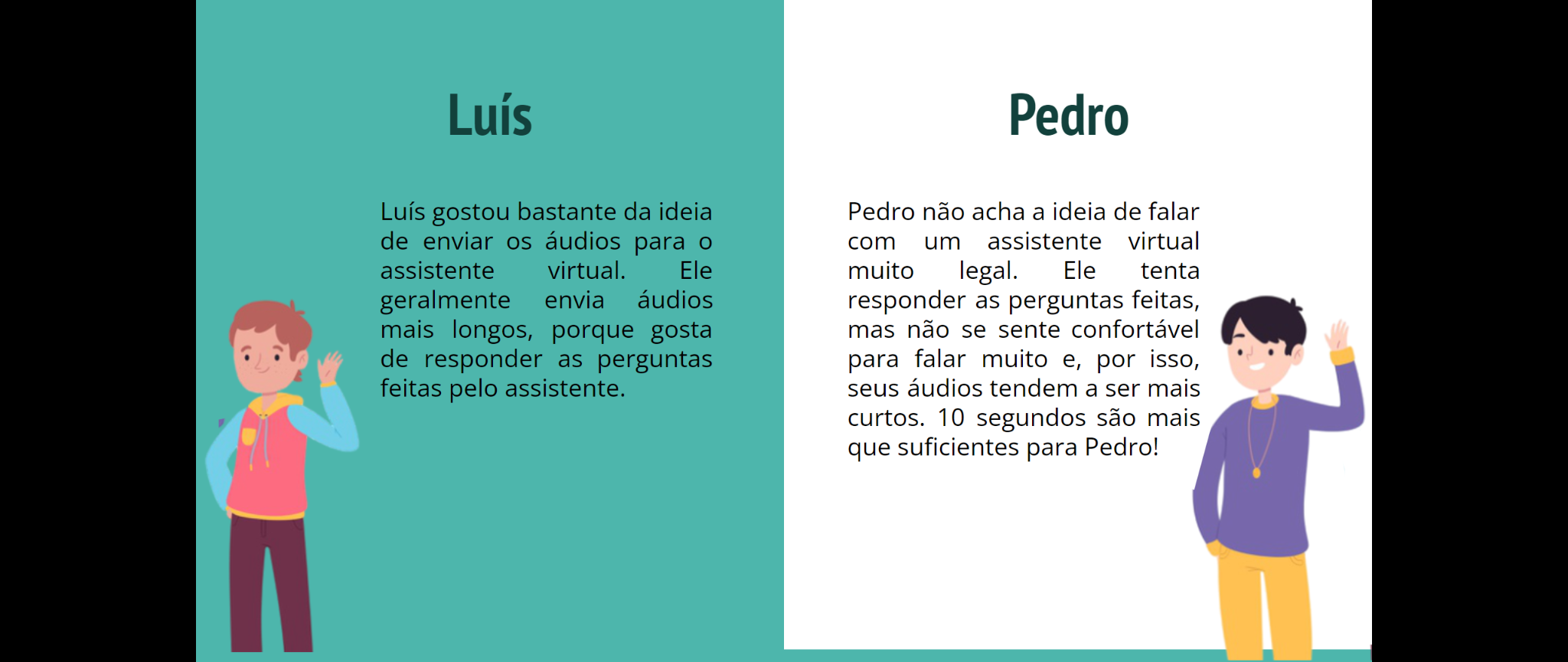


Luís really liked the idea of sending the audios to the virtual assistant. He usually sends longer audios because he likes to answer the questions asked.

Pedro doesn't think the idea of talking to a virtual assistant is very cool. He tries to answer the questions, but he doesn't feel comfortable talking too much, so his audios tend to be shorter. 10 seconds is more than enough for Pedro!

Scenario 2 - Time of the first daily interaction


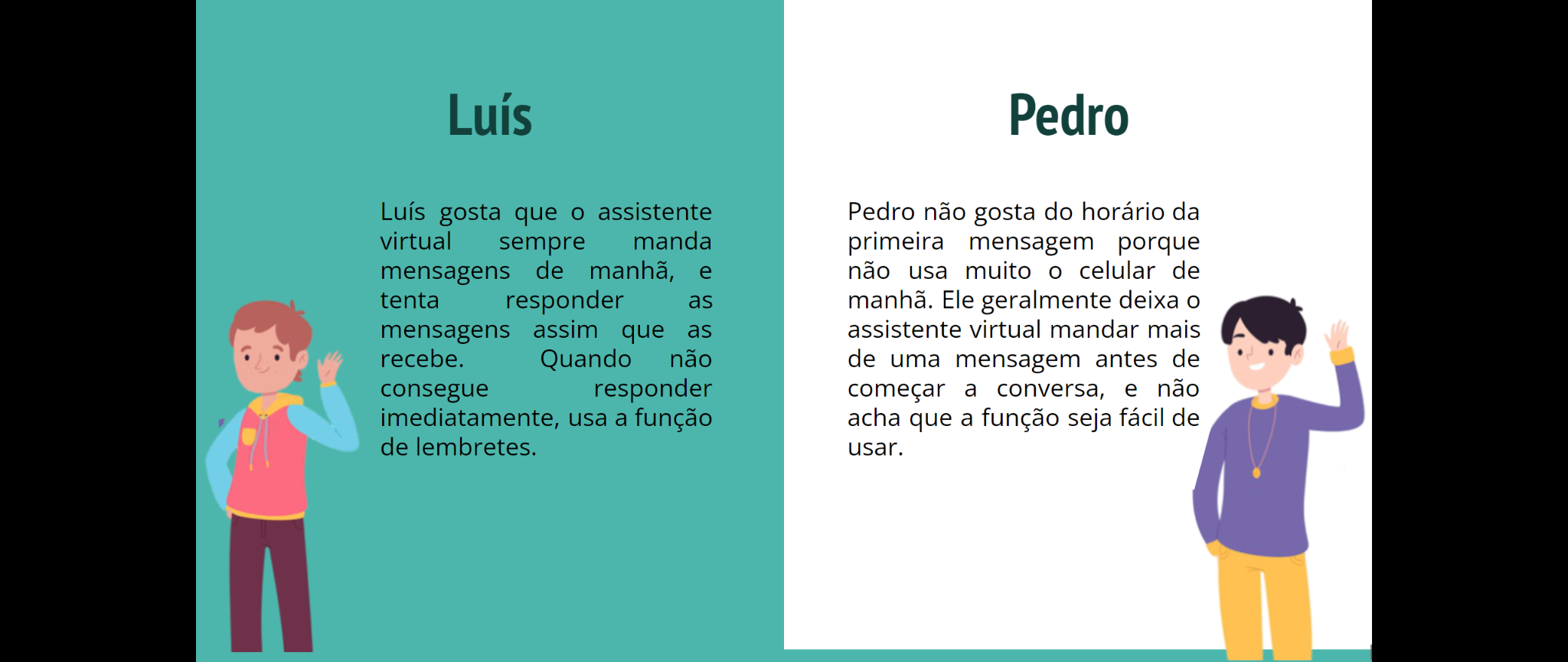


Luís likes it that the virtual assistant always texts in the morning, and tries to respond to messages as soon as he receives them. If he can't respond right away, he uses the reminders function.

Pedro doesn't like the time of the first message because he doesn't use his cell phone much in the morning. He usually lets the virtual assistant send more than one message before starting the conversation, and he doesn't find the snooze function easy to use.

Scenario 3 - Frequency of messages
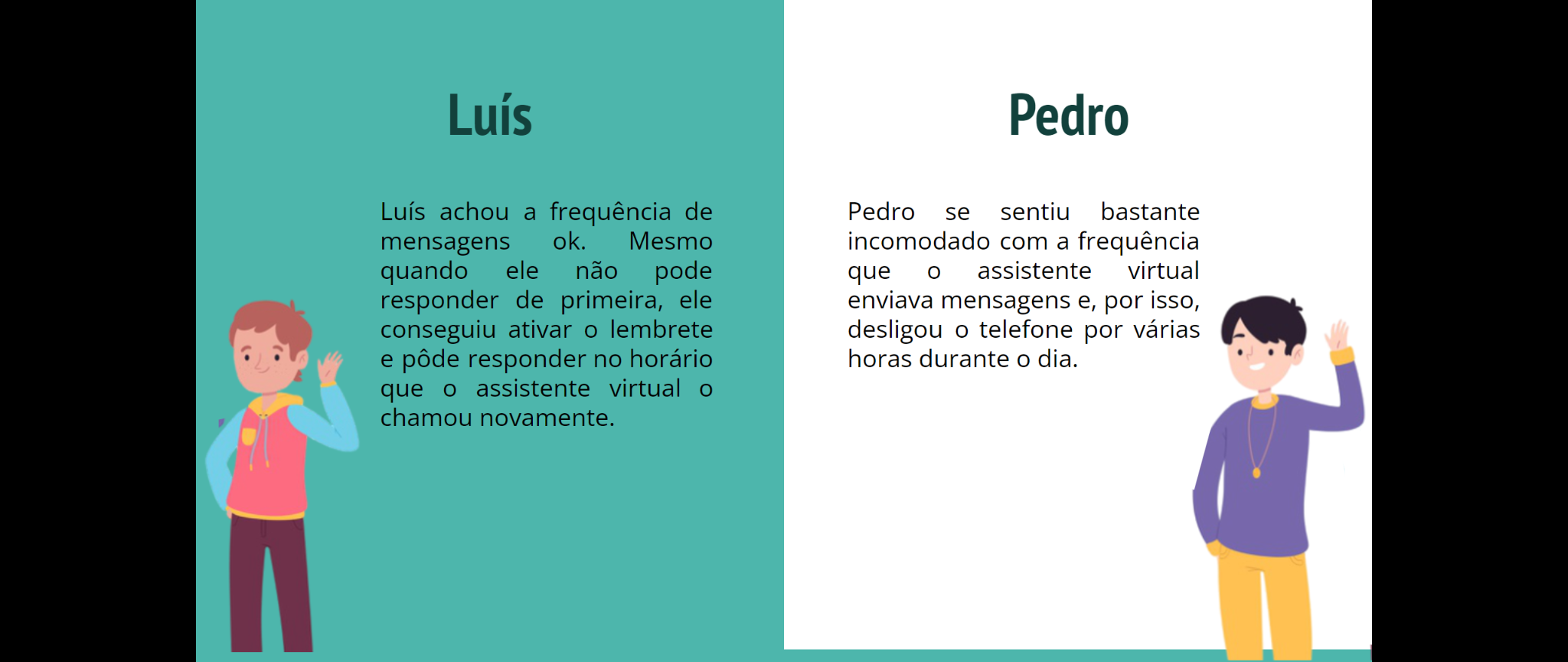


Luís found the frequency of messages ok. Even when he couldn't respond at first, he was able to activate the snooze function and was able to respond at the time the virtual assistant called him again.

Pedro felt quite annoyed with how often the virtual assistant sent messages, so he turned off the phone for several hours during the day.

Scenario 4 - MFQ answers


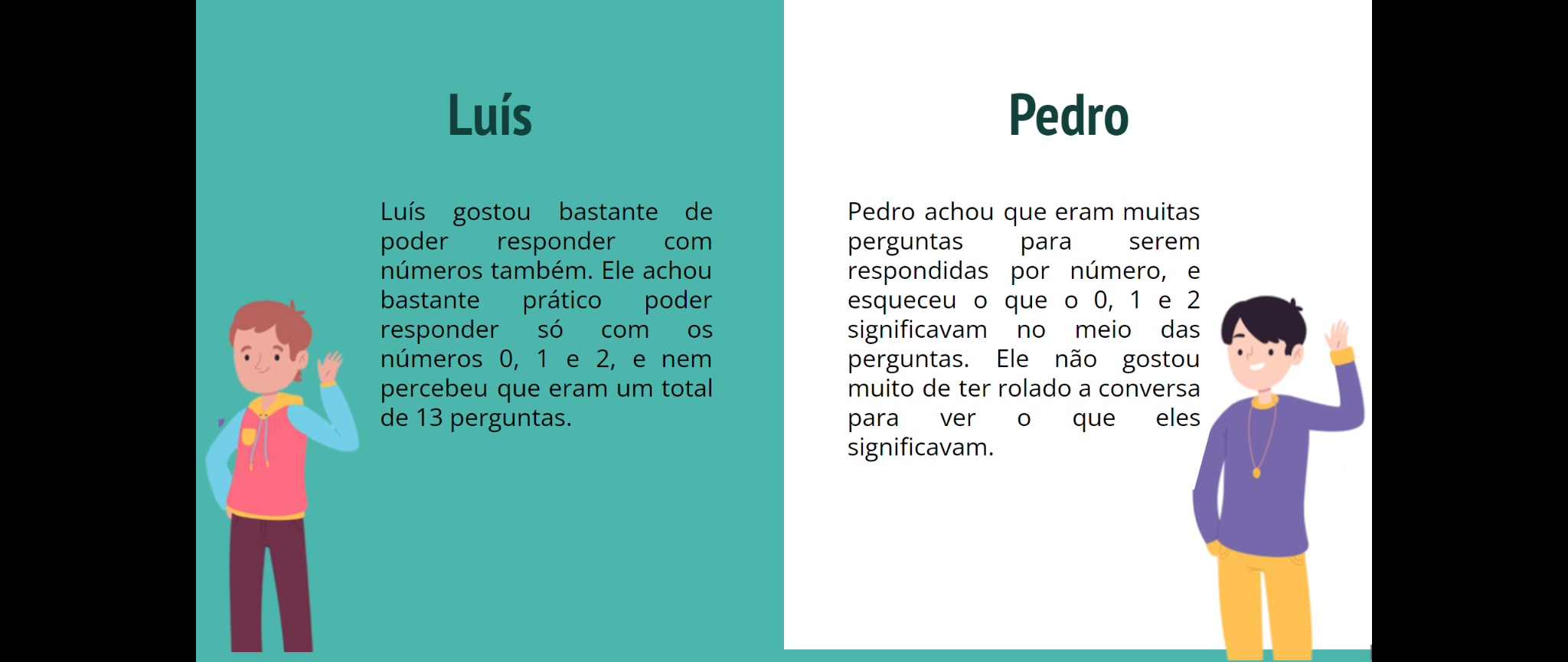


Luís really liked answering with numbers too. He found it very practical to answer only with the numbers 0, 1 and 2, and he didn't even realize that there were a total of 13 questions.

Pedro thought that there were too many questions to be answered by number, and he forgot what the 0, 1 and 2 meant in the middle of the questions. He didn't much like having to scroll the conversation to see what the numbers meant.

Scenario 5 - Overall acceptability of IDEABot


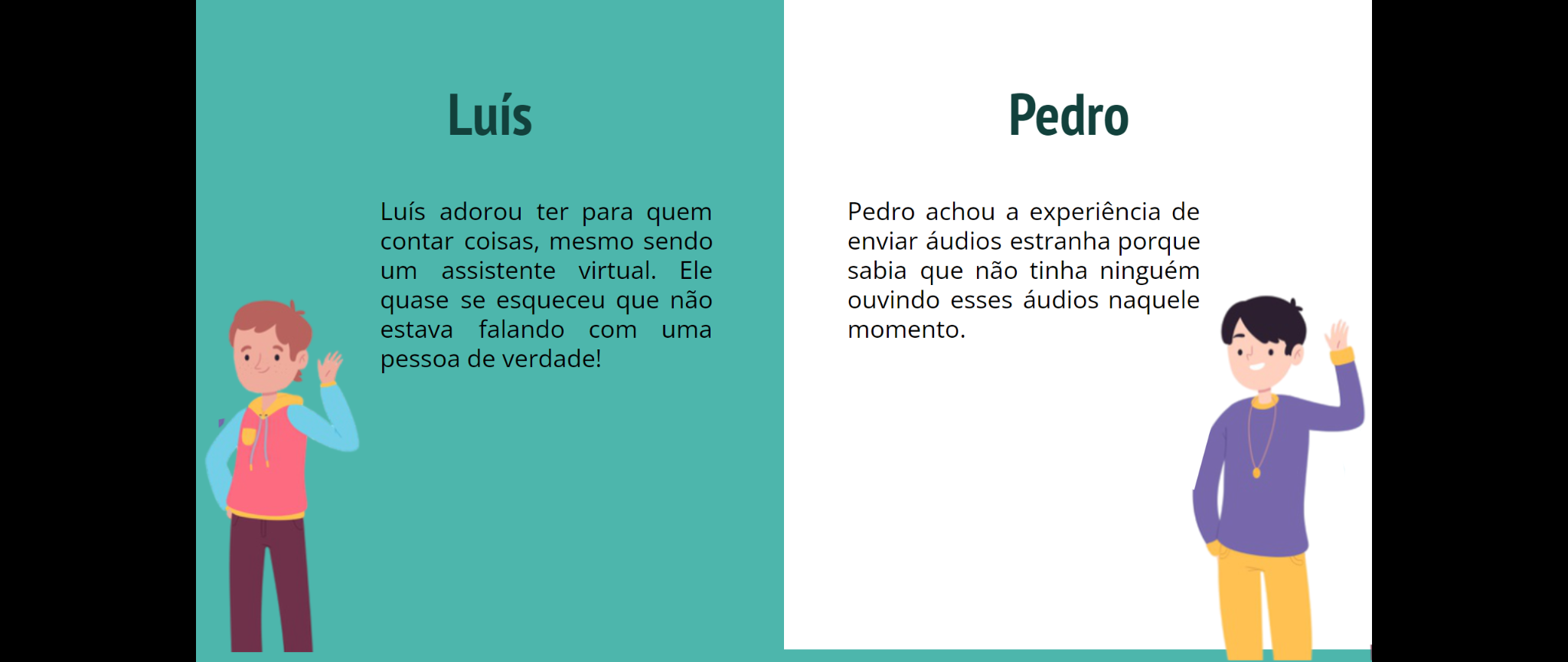


Luís loved having someone to tell things to, even though it was a virtual assistant. He almost forgot he was talking to a bot, and not to a real person!

Pedro found the experience of sending audios strange because he knew that no one was listening to these audios at the moment when they were sent.

Scenario 6 - Feasibility of 15 day-collection period


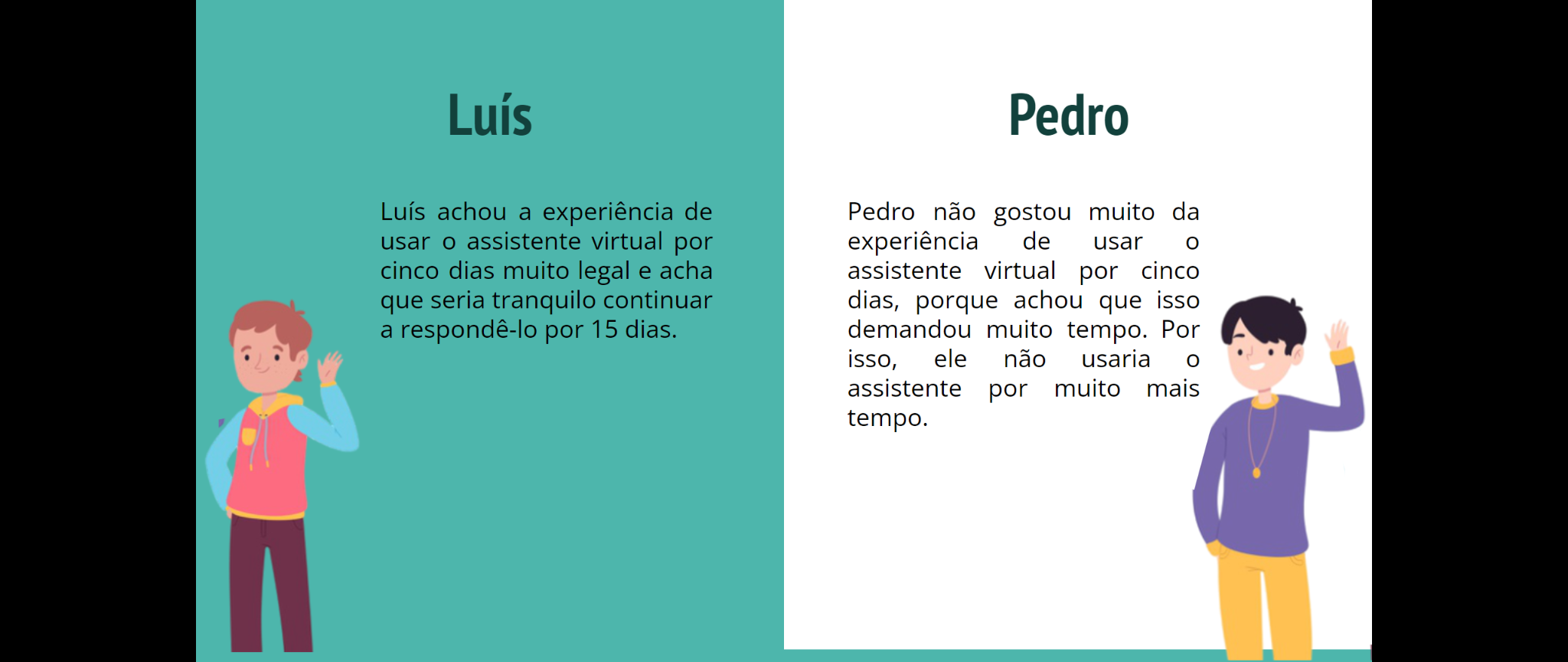


Luís found the experience of using the virtual assistant for five days very cool and thinks it would be easy to continue to answer for 15 days.

Pedro didn't really like the experience of using the virtual assistant for five days, because he felt that it took too much time. So he wouldn't use the assistant for a longer period.
