# Supplementary material for: Assessing Mood With the Identifying Depression Early in Adolescence Chatbot (IDEABot): Development and Implementation Study
Source: JMIR Hum Factors. 2023 Aug 7;10:e44388. doi: 10.2196/44388 (PMC10442728; doi:10.2196/44388)
Supplement: Multimedia Appendix 3 [file humanfactors_v10i1e44388_app3.docx]

**Supplementary file for “Assessing Mood With the Identifying Depression Early in Adolescence Chatbot (IDEABot): Development and Implementation Study”**

**Multimedia Appendix C -** IDEABot’s types of interaction

*First Interaction*

IDEABot’s first interaction with users (D0) aims at explaining tasks and functions. This is subsequent to participants receiving the animated video explaining the goal of data collection and information on measures taken to ensure data confidentiality. After receiving the video, participants also completed the consent form. In the D0, all types of answers to be requested in the subsequent days are tested, also indicating the format in which they will be requested (e.g., text or audio). The introduction also makes sure that participants are aware of the nature of the interactions, i.e., that they understand the bot is not a person, and also that when audios are collected they will not be heard immediately. In this first interaction, the chatbot asks adolescents to state their names, and this user input is stored and used for future interactions. After explaining its functioning, IDEABot asks a question requiring an audio answer (“What have you done today? What else will you do during the day?”). Since this interaction was of obligatory completion and had the goal of training users to interact with the IDEABot, there were no probing questions or ideal total duration for the audio question. Thus, we chose to exclude D0 in the evaluation of user engagement and other analyses. The reminder ("snooze") function is also explained.

*Daily Interactions*

*Mood ratings*

On all 15 days, participants were asked to rate their mood on a Likert-type scale ranging from 0 (very sad) to 10 (very happy), with 5 as a neutral point. They were instructed to type an integer number — the chatbot probes participants until they provide an acceptable value.

*Emoji mood rating*

After being asked to rate their mood from 0 to 10, participants were also asked to equate their numeric answer with one of five emojis (😭🙁😐🙂😁). This rating was also requested on all 15 days of interaction. Of note, WhatsApp standardized emojis regardless of the phone’s operating system. However, in older versions of Android phones, emojis may appear according to the phone’s system. The IDEA-RiSCo sample recruitment criteria had no specific requirement beyond having a working phone with WhatsApp access. Therefore, participants with older versions of WhatsApp installed (prior to 2017) might not have used exactly the same emoji keyboard as intended in the IDEABot’s design, but the equivalent in Android’s emoji keyboard.

*Brief audio recordings*

On days 1, 2, 4, 6, 8, 10, 12, and 14, participants were requested to respond to questions on their routine, social interactions, and preferences using audio recordings. The chatbot aimed to collect at least 1 minute of audio each day; it was designed to probe participants up to four times until the total audio duration for the interaction reaches at least 1 minute. Nonetheless, participants are given the option to deny sending further audio messages after the third probing question, in which case the bot would end the day’s interaction.

*Mood and Feelings Questionnaire - Short Version*

On days 3, 5, 7, 9, 11, 13, and 15, participants were asked to respond to the 13 questions of the Short Mood and Feelings Questionnaire (sMFQ), Brazilian version. Possible sMFQ answers are "not true" (0), "sometimes" (1), or "true" (2). Participants were asked to type only the number corresponding to their answer. Although the original sMFQ asks participants about the past 2 weeks, we adapted the tool in the present protocol asking participants to respond about their current day.

*Other messages*

The IDEABot is also programmed to respond to unprompted messages. Participants can send spontaneous audios, to which the chatbot will respond “Hi, {participant}! Thank you for sending another audio! If you want to say anything else, you can send audios at any time”. If participants send other, unsupported media (e.g., text, video, photos), the bot responds by asking for an audio message (“{participant}, it’s great seeing you using this space! It would be important if you sent me this in audio format. Could you send me an audio explaining what you just sent me?”).

*“Snooze” function*

The IDEABot is set to start sending daily prompts at 1:30 PM, asking participants about their availability to answer the questions for the day. If participants do not interact with the bot until 2 PM, or if they inform the bot that they are not available to answer, IDEABot suggests setting a reminder, i.e., a time considered more convenient to the user to respond to the day’s interaction. Additionally, if participants stop responding for more than 30 minutes in the middle of interactions, the IDEABot also suggests setting a reminder.

Participants can choose to enable the “snooze” function throughout all interactions. The IDEABot uses a 24-hour format, usual in Brazil, which enables participants to set reminders at any time until 3 AM the next day. In this format, AM and PM are easily distinguishable, and participants are instructed to provide time in the format HH:MM (15:30 for 3:30 PM, for example). If participants send an unsupported format, the IDEABot sends an error message (“It seems like you would like to set a reminder. But I need you to use a format that I understand: it can’t be a time that has already passed, neither after 3 AM”).
