# Supplementary material for: Assessing Mood With the Identifying Depression Early in Adolescence Chatbot (IDEABot): Development and Implementation Study
Source: JMIR Hum Factors. 2023 Aug 7;10:e44388. doi: 10.2196/44388 (PMC10442728; doi:10.2196/44388)
Supplement: Multimedia Appendix 4 [file humanfactors_v10i1e44388_app4.docx]

**Supplementary file for “Assessing Mood With the Identifying Depression Early in Adolescence Chatbot (IDEABot): Development and Implementation Study”**

**Multimedia Appendix D -** Chatbot script for the Mood and Feelings Questionnaire - Short version (sMFQ) instructions

We now want to know how much the following statements apply to your day today.

Please answer by typing numbers: for each question, type only one number, that is, 0, 1 or 2 .

For example: “I felt very sad”.

If you type 0, it means that this does not apply to you today - you are not feeling sad today.

If you type 1, it means that this applies to you today to a certain extent - you are feeling just a little sad today, or you are feeling sad during certain moments.

If you type 2, it means that this applies to you today - you are feeling sad most of the time today.

Finally, if you want to change the last answer you entered, just type “revise”.
