# Supplementary material for: Assessing Mood With the Identifying Depression Early in Adolescence Chatbot (IDEABot): Development and Implementation Study
Source: JMIR Hum Factors. 2023 Aug 7;10:e44388. doi: 10.2196/44388 (PMC10442728; doi:10.2196/44388)
Supplement: Multimedia Appendix 5 [file humanfactors_v10i1e44388_app5.docx]

**Supplementary file for “Assessing Mood With the Identifying Depression Early in Adolescence Chatbot (IDEABot): Development and Implementation Study”**

**Multimedia Appendix E -** Processing and analysis of data and questionnaires

The IDEABot was designed to collect at least 7 minutes of audio per participant at the end of the 15-day cycle. This goal was designed excluding the first day of interaction with the bot (D0). Two types of responses associated with audio data were stored. First, audio metadata (total duration of audio, audio timestamp, and total time between the bot’s prompt and participant’s answer) were stored in the relational database that aggregates the textual interaction data under the participant’s ID. Audio files were stored and uploaded into Dropbox organized by the correspondent prompt. Audio files were initially stored under the participant’s phone number, which was later pseudo-anonymized into the participant’s ID. For each prompt, a folder containing the corresponding audio was created. Later processing included linking the audio files to the corresponding metadata in the relational database.

Audio recordings enable analyses of both content and acoustic features. As the first step prior to analyses, all audio recordings were transcribed. Transcription was performed manually by two researchers independently, with disagreements resolved by a third researcher. While the use of voice recognition software is steadily increasing, the reliability of these tools is still limited, especially for audio recordings which are not in English. Nonetheless, audio transcription is a vital step for analysis of audio data, and inadequate preparation of transcripts can delay or negatively affect the analysis process. Therefore, we aimed to establish a transcription protocol that focused on preserving the morphologic naturalness of the transcription (i.e., maintaining the word forms) and generating verbatim accounts. Moreover, our goal was to make transcripts suitable for both researcher use in qualitative approaches and computer-based analyses.

To that, a transcription cover sheet was developed to compile information regarding transcriber name, date of transcription, participant ID, and total number of audio files recorded. Because each transcription included several audio files, we also created a header-sheet for each audio, including audio file name, total duration, number of interlocutors and presence of other sounds (such as music) in the background. Each information was coded manually by two independent transcribers, who also verified and reported whether the audios were blank. After independent transcription, all transcripts were manually compared to identify discrepancies by one of the researchers, which if present was resolved by a third researcher. In the transcription process, names and/or information that could point to an identification of the speaker were identified and signaled using double brackets. After the comparison process, this information was deleted from the final version of the transcripts that would be later used for analyses. Additional methodological steps involved deleting third-party dialogues recorded by the participants (such as parallel talks and interactions with others), and sound interferences that make the voice inaudible. The final version of each transcript was stored in a secure server provided by Hospital de Clínicas de Porto Alegre.

Further to text transcripts, acoustic features from audio recordings, as well as techniques such as speech recognition can be used to automatically extract features related to prosody, tone of voice, and intensity. In these analyses, the main focus of interest is on the acoustic characteristics of the audio — such as wave amplitude values over time or transformation of audios into melspectograms (images that inform about the predominant frequencies in certain portions of the audio). Future analyses will leverage previously annotated databases in Brazilian Portuguese — several of which are currently available. The selection of traits of interest and of a database for emotional recognition is one of the most important analytic stages, and will require ascertainment of the quality of audios recorded by participants and of the audio data obtained. Analyses focusing on defining and extracting fundamental frequencies (vibration produced by vocal folds, essential for classifying and characterizing a voice signal) will allow categorization of voiced and unvoiced audios, as well as extraction of other relevant features.

Nonetheless, it is important to emphasize the complementarity of textual and acoustic aspects obtained from audio recordings: the recognition of emotions from text (either provided by the user or extracted from audio transcriptions) is often dependent on the content of the audio, relying on the user’s mentioning of keywords that will be later tagged as indicative of emotions. In turn, recognition of emotions from sound allows an independent assessment of content, relying on acoustic features (such as identification of the frequency of sound waves) to infer emotional states even when speech is incomprehensible. Therefore, combining both approaches may be ideal for understanding the full range of information that can be conveyed and extracted from audio recordings.

In addition to daily self-reported mood data, IDEABot collected and stored all conversation logs in the form of a relational database, i.e., a database that stores data points that are related to one another, under each participant's ID. The chosen database manager was PostgreSQL running on a virtual private server. For each interaction, in addition to the textual responses to the bot's prompts, IDEABot also stored metadata related to each answer. These included: timestamp of each response, total duration of each interaction with the bot (total time between first and last answer), and number of times participants used the "snooze" function. Additionally, it stored the corresponding prompt for each conversation, in order to match responses.

For the sMFQ responses, metadata was also collected and stored for each sMFQ response. All individual answers were stored even when participants did not complete the whole questionnaire. Additionally, full conversation logs were stored at the smartphones where the WhatsApp interface was installed, and a REDCap backup hosted at Hospital de Clínicas de Porto Alegre stored all self-reported data on mood.
